# COVID-19 vaccination uptake in Belgium: socioeconomic and sociodemographic disparities

**DOI:** 10.1093/eurpub/ckac129.046

**Published:** 2022-10-25

**Authors:** L Cavillot, J Van Loenhout, L Catteau, L Van den Borre, R De Pauw, K Blot, N Speybroeck, B Devleesschauwer, P Hubin

**Affiliations:** 1 Epidemiology and Public Health, Sciensano, Brussels, Belgium; 2 Research Institute of Health and Society, University of Louvain, Brussels, Belgium; 3 Interface Demography, Free University of Brussels, Brussels, Belgium; 4 Rehabilitation Sciences, University of Ghent, Ghent, Belgium; 5 Department of Translational Physiology, Infectiology and Public Health, University of Ghent, Merelbeke, Belgium

## Abstract

**Background:**

Recent studies have identified important social inequalities in SARS-CoV-2 infection and related COVID-19 outcomes in the Belgian population. This study aims to investigate socioeconomic and -demographic characteristics associated with the uptake of COVID-19 vaccine in Belgium.

**Methods:**

We conducted a retrospective analysis of the uptake of the first dose of COVID-19 vaccine among 5,341,584 adults (≥ 18 years) tested for COVID-19 in Belgium until August 31, 2021. We integrated four national data sources: the Belgian vaccine registry (vaccination status), COVID-19 Healthdata (laboratory test results), STATBEL (socioeconomic/-demographic data) and the Common Base Registry for HealthCare Actors (people licensed to practice a healthcare profession in Belgium). Unvaccinated and vaccinated people (with at least one dose) were compared using multivariate logistic regression analysis.

**Results:**

During the study period, 53,887 people (10%) did not receive the first COVID-19 vaccine dose in Belgium. Migrant background was associated with vaccine uptake (e.g., non-Europeans were almost three times [2.96-3.00] more likely to be unvaccinated compared to Belgian nationals). Single parents (OR 1.27 [1.26-1.28]) and people living alone (OR 1.18 [1.17-1.19]) were more likely to be unvaccinated compared to couples with children. Having a low or moderate education level (OR 1.36 [1.35-1.38] for low; OR 1.30 [1.29-1.32] for moderate) and income (OR 2.36 [2.34-2.38] for low; OR 1.54 [1.52-1.55] for moderate), being unemployed, (OR 1.50 [1.49-1.51), and having low health literacy (OR 1.41 [1.39-1.43]) led to a greater likelihood of being unvaccinated.

**Conclusions:**

Migrants, people living alone, single parents or socioeconomically disadvantaged groups have lower uptake of COVID-19 vaccine in Belgium. The identification of these socioeconomic and -demographic disparities is critical to develop strategies guaranteeing a more equitable COVID-19 vaccination coverage in Belgium.

**Key messages:**

• The study highlight important determinants in the uptake of the first dose of COVID-19 vaccine in Belgium.

• These results highlight the importance to focus efforts on socioeconomically disadvantaged groups currently under-represented in COVID-19 vaccination uptake in Belgium.

